# Structure–Function Relationship of Novel Tetrakis (Mercapto-Terphenyl)Benzene Cobalt (II) Phthalocyanines: Synthesis and Computational Evaluation

**DOI:** 10.3390/molecules30132693

**Published:** 2025-06-22

**Authors:** Sevil Sener, Nursel Acar-Selcuki

**Affiliations:** 1 Department of Chemical Technology Program, Aliaga Vocational School, Ege University, Izmir 35040, Türkiye; 2 Department of Chemistry, Faculty of Science, Ege University, Izmir 35100, Türkiye; nursel.acar@ege.edu.tr

**Keywords:** metallophthalocyanine, thiol, terphenyl, density functional theory

## Abstract

This study introduces a newly synthesized Co(II) phthalocyanine complex (Co-Pc, **4**) incorporating two (mercapto-terphenyl)thio-dione substituents, along with a detailed exploration of its structural, spectroscopic, and binding characteristics. The key precursor, 4-[(4′′-mercapto-[1,1′:4′,1′′-terphenyl]-4-yl)thio]phthalonitrile (compound **3**), was first obtained and subsequently used to construct the phthalocyanine macrocycle through cyclotetramerization in the presence of cobalt and zinc salts under heat and vacuum in dimethylformamide. The resulting compounds (**3** and **4**) were characterized using a comprehensive array of analytical techniques, including elemental analysis, UV–Vis spectroscopy, FT-IR, ^1^H-NMR, and Q-TOF mass spectrometry. Additionally, density functional theory (DFT) and time-dependent DFT (TD-DFT) calculations were employed to elucidate the electronic structure and geometrical features of Co-Pc **4**, providing theoretical support for the experimental findings. The integration of theoretical and experimental findings provides in-depth insight into the electronic behavior and reactivity of compound **4**, highlighting its promise as a candidate for photovoltaic applications. Further studies may investigate how structural modifications influence these properties, potentially leading to improved device performance.

## 1. Introduction

Phthalocyanines are synthetic macrocyclic compounds first discovered by accident in 1907. In this study, cobalt(II) was chosen as the central metal due to its well-documented redox activity, coordination versatility, and established applications in electrocatalysis and sensing. The use of sulfur-rich, π-extended terphenyl-substituted ligands is expected to enhance intramolecular charge transfer and reduce the HOMO–LUMO gap, potentially offering superior optoelectronic performance compared to oxygen-substituted analogs. Two decades later, in 1927, Diesbach and colleagues synthesized the remarkably stable copper phthalocyanine complex. The structural elucidation of phthalocyanines, which began around 1929, was finalized and reported in 1934. Since then, both metal-free and metal-coordinated variants of phthalocyanines have been widely synthesized and investigated. Due to their exceptional thermal and chemical stability, phthalocyanines have been utilized as dyes and pigments in inks, textiles, metals, and plastics. Beyond traditional uses, they have gained prominence in high-tech applications including photoconductivity, photodynamic therapy, and chemical sensing. They are also employed in laser dyes, electrochromic displays, optical storage media, photovoltaic cells, liquid crystals, olefin oxidation catalysis, fuel enhancement, and nonlinear optics (NLO) and optical limiting (OL) technologies [1,2,3].

In addition to conventional phthalocyanines, advanced structures such as polymeric forms, binuclear sandwich and clamshell types, and multi-nuclear complexes (tri-, tetra-, penta-, and octa-nuclear) have been reported. In 2002, the Tomilova group introduced a novel class of phthalocyanines known as “top-type” phthalocyanines. Following this breakthrough, the Bekaroğlu group expanded the structural diversity of top-type phthalocyanines through the introduction of various bridging units. These modified top-type phthalocyanines have demonstrated properties such as gas sensing, electrochemical and electrochromic behavior, as well as NLO and OL effects. The specific nature of the bridging ligands significantly influences the performance and application potential of these compounds, highlighting the continued importance of phthalocyanine synthesis [4,5].

The present study focuses on the synthesis of novel phthalocyanine derivatives containing extended π-conjugated systems through the incorporation of three aromatic rings via the precursor 1,4-bis(4-mercaptophenyl)benzene (TPDT). Following synthesis and structural elucidation, the redox behavior of these new phthalocyanines will be investigated. Complementary computational studies using density functional theory (DFT) will be employed to optimize molecular geometries and calculate key electronic and thermodynamic parameters. Sulfur-containing aromatic phthalocyanines are of particular interest due to their potential application in electronic systems, notably as electrolyte materials in lithium-sulfur batteries [6].

Lithium-sulfur batteries are known for their lightweight design, robustness against thermal and mechanical stress, reusability, deep discharge capacity, long shelf life, and eco-friendliness. These features make them especially suitable for defense technologies, electric vehicles, mobile devices, and solar energy storage [7,8,9]. Moreover, thiol-functionalized compounds often exhibit notable biological activity, rendering them potential candidates for photodynamic therapy (PDT) and boron neutron capture therapy (BNCT), as demonstrated in our previous work [10,11].

In this context, the Co(II) phthalocyanine (Co-Pc) complexes derived from TPDT were subjected to a full range of characterization techniques, including elemental analysis, UV–visible spectroscopy, FT-IR, ^1^H-NMR, and Q-TOF mass spectrometry. Subsequently, DFT and TD-DFT methods were applied to model the structural and electronic features of the phthalocyanines and to simulate their UV–Vis spectra. These theoretical predictions were benchmarked against the experimental spectra recorded in DMSO, providing comprehensive insight into the optoelectronic behavior of the synthesized compounds and their potential for advanced technological and biomedical applications.

## 2. Experimental

### 2.1. Characterization Techniques

Structural characterization and elemental composition analyses of the synthesized compounds were conducted using a CHNS-932 elemental analyzer (LECO, St. Joseph, MI, USA). The ^1^H nuclear magnetic resonance (^1^H-NMR) spectra were recorded on a Varian-Innova 400 MHz spectrometer, with chemical shifts referenced to tetramethylsilane (TMS, Si(CH_3_)_4_) as the internal standard. UV–Vis absorption spectra were measured using a PerkinElmer Lambda 35 spectrophotometer in 1 cm path-length quartz cuvettes, while FT-IR spectra were obtained on a PerkinElmer Fourier transform infrared spectrophotometer.

High-performance liquid chromatography (HPLC) analyses were performed using an Agilent 1260 Infinity system (Agilent Technologies, Santa Clara, CA, USA) equipped with dual pumps, a degasser, and an autosampler. Chromatographic separation was achieved using a Poroshell 120 EC-C18 column (4.0 × 60 mm, 2.9 μm particle size; Agilent). The mobile phase consisted of 0.2% acetic acid in water (solvent A) and acetonitrile (solvent B), with a gradient elution profile as follows: 0–0.6 min, 6% B; 0.6–9 min, 16% B; 10–12 min, 21% B; 12–15 min, 26% B; 15–19 min, 31% B; 19–22 min, 46% B; 22–25 min, 61% B; 25–29 min, 81% B; and 29–31 min, 91% B, followed by re-equilibration at 6% B for 3 min. The column temperature was maintained at 36 °C, the injection volume was set to 4 μL, and the flow rate was 0.6 mL/min.

Mass spectrometry (MS) analysis was carried out using an Agilent 6550 iFunnel QTOF-MS system equipped with a Dual AJS ESI (electrospray ionization) source, operating in positive ion mode. Instrument settings included a drying gas temperature of 295 °C at a flow rate of 15.0 L/min, nebulizer pressure at 40 psi, capillary temperature at 400 °C, and a collision cell nitrogen flow rate of 15 L/min. Mass spectra were acquired over an *m*/*z* range of 60–2050.

### 2.2. Synthesis Studies

**Scheme 1:**
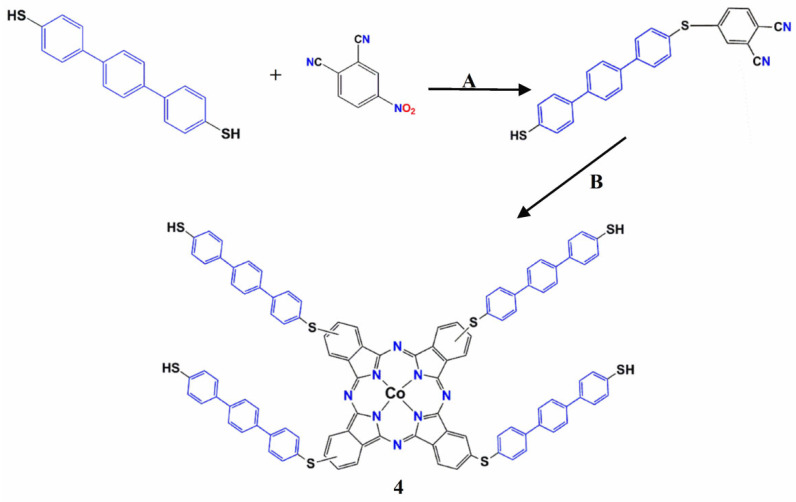
Synthesis of 3,4 (i) Cs_2_CO_3_, N2, ACN, 80 °C, 72 h; (ii) Co(OAc)2∙4H2O, 220 °C, DMF, N2, 24 h.

The synthesis of compound **4**, namely 2,9(10),16(17),23(24)-tetrakis-[2-(E)-(2-(E)-2-mercaptobenzylidene)mercaptoterphenyl]-2-thio cobalt(II) phthalocyanine, is illustrated in Scheme 1. Compound **4** was obtained via a cyclotetramerization reaction of 4-[(4″-mercapto-[1,1′:4′,1″-terphenyl]-4-yl)thio]phthalonitrile **(3)**, which was synthesized from 1,1′:4′,1″-terphenyl]-4,4″-dithiol (or alternatively from 1,4-bis(4-mercaptophenyl)benzene) **(1)** and 4-nitrophthalonitrile **(2)**.

#### 2.2.1. Synthesis of Phthalonitrile (**3**) 4-[(4′′-Mercapto-[1,1′:4′,1′′-Terphenly]-4-Yl)Thio) Phthalonitrile

Anhydrous Cs_2_CO_3_ (1.0 g, 8.0 mmol) and 1,4-Bis(4-mercaptophenyl)benzene (TPDT) 1 (0.2 g, 1.0 mmol) and 4-nitrophthalonitrile 2 (0.36 g, 2.0 mmol) were dissolved in acetonitrile (ACN) (25 mL). This solution was refluxed at 80 °C for 72 h under a nitrogen atmosphere. The resulting solution was cooled to room temperature, and Cs_2_CO_3_ was removed by filtration. The solvent of the obtained solution was evaporated under vacuum to yield a solid. This solid was washed with petroleum ether and purified using column chromatography. Thin-layer chromatography (TLC) was used to guide the solvent selection. Despite multiple eluents being tested, impurity removal required polarity variation. We acknowledge that reversed-phase chromatography may offer a more sustainable approach using various polar and non-polar solvents such as acetone, tetrahydrofuran (THF), dimethyl sulfoxide (DMSO), dimethylfuran, and chloroform. Yield: 0.26 g (78%). Mp. 114 °C; Anal. calculated for C26H16N2 S2 (M.W. 420.08 g/mol): C, 74.25; H, 3.83; N, 6.66; S, 15.25%, found C, 78.80; H, 3.50; N, 13.30; %; 1H NMR (400 MHz, acetone-d6) δ ppm) 7.76–7.66 (d, 1H,) (s, 1H, CH); 73.30–2.48 (d, 1H) (s, 1H, SH).

^13^C NMR (DMSO-*d*_6_ *δ* ppm): (135.6, Ar-C-SH); (128.98, Ar-C-C). IR (KBr): *ν*_max_/cm^−1^ 3484 and 3390 (H2O), 3094 (Ar-CH), 2220 (S-H), 2160 (Ar-C≡N), 1726, 1664, 1581, 1477 (Ar-C=C), 1386, 1215, 1132 (Ar-C-N), 1189, 1045, 868, 810 (S-H), 751, 720, 692 (C-S). MS *m*/*z*: 420.08 (M+, 100%) (C_22_H_10_N_2_ O_4_) and 421.14 (M+H) ^+^.

#### 2.2.2. Synthesis of Compound (**4**) 2, 9(10), 16(17), 23(24)-Tetrakis-(2-(E-(2-(E-2-Mercaptobenzyliedene) Mercaptoterphenly-2-Thio Cobalt(II)

Nitril TPDT 3 (0.10 g, 0.30 mmol) and Co(AcO)2∙4H2O (0.02 g, 0.08 mmol) were dissolved in DMF and heated in a sealed Schlenk tube under nitrogen atmosphere at 220 °C, which allows reactions above the boiling point of DMF under nitrogen atmosphere in an isolated Schlenk flask. The mixture was then heated at 220 °C for 24 h. After the reaction was cooled to room temperature, a green-black precipitate was observed. This product was then purified by washing with hot DMF, THF, ethyl acetate, chloroform, ether, ethanol, and water, in sequence. Finally, it was dried in an oven at 105 °C. The product dissolved in hot DMSO. Yield: 0.02 g (22%). Mp. 218 °C; Anal. Calculated for C104H64N8S8Co (M.W.: 1741.13 g/mol): C, 71.74; H, 3.70; N, 6.44; S, 14.73%, found C, 68.41; H, 4.22; N, 7.49; S, 15.76%. UV–Vis (DMSO): λmax/nm (log ε): 691(4.9). IR (KBr, ν_max_/cm^−1^): 3378 (H2O), 2890 (Ar-CH), 1714 (Ar-C=N), 1600 (Ar-C=C), 1436 (Ar-C-N), 1377, 1035, 1001 (Ar-C=N), 815 (S-H); 739 (C-S). MS *m*/*z*: 1741.13 (M+, 100%) (C104H64N8S8Co ∙4H2O) and MS: *m*/*z* 1739.20 [M-H]+(calc. *m*/*z* 1741.13 [C104H64 Co N8S8].

Compound **3** is a mononitrile derivative bearing extended aromatic substitution through terphenyl-thiol linkages. The structure provides high electron delocalization and multiple coordination sites for metal chelation.

### 2.3. Computational Details

Optimizations of the studied systems were carried out using Gaussian 16 [12] in gas phase and in dimethyl sulfoxide (DMSO). Molecular structures, UV–Vis spectra, and orbitals were visualized using Gaussview 6.0 [13] and Avogadro programs [14]. Density functional theory (DFT) [15,16] calculations were carried out using the dispersion-corrected B3LYP level of theory (B3LYP-D3/6-31G(d)) [17]. Geometry optimizations were performed at the ground state. The hybrid B3LYP functional—comprising Becke’s three-parameter nonlocal exchange functional (B3) [18] and the Lee–Yang–Parr correlation functional (LYP) [19]—was employed for the DFT calculations. Time-dependent density functional theory (TD-DFT) [20] calculations were performed using the 6-31+G(d) basis set [21]. The true minimum nature of the optimized structures was confirmed by the presence of all positive vibrational frequencies. Excited-state properties were evaluated by calculating the first 120 singlet excited states for the investigated systems. Molecular orbital energies and UV–Vis spectra were determined based on ground-state geometries. Solvent effects were investigated at the ground state using the polarizable continuum model (PCM) [22,23], as implemented in Gaussian 16.

## 3. Results and Discussion

### 3.1. Chemical Synthesis Studies

Compound **3** was synthesized with an overall yield of 78%. During optimization, various reaction temperatures (180–200 °C) and durations (12–18 h) were tested. However, these conditions led to lower yields or impure products. Optimal results were achieved at 220 °C for 24 h. The novel phthalocyanine derivative, compound **4**, was subsequently obtained via cyclotetramerization using the corresponding metal acetates Co(OAc)2·4H2O in anhydrous dimethylformamide (DMF), as illustrated in Scheme 1. Detailed synthetic procedures and characterization data are presented in the synthesis section.

The FT-IR spectrum of compound **3** (Figure S1) displays a sharp and distinct peak at 2229 cm^−1^ corresponding to the (Ar–C≡N) stretching of the mononitrile functional group stretching vibration [3], confirming the successful synthesis of the mononitrile precursor. Additional characteristic peaks observed at 3094 cm^−1^ (Ar–CH), 1726, 1664, 1581, and 1477 cm^−1^ (Ar–C=C), along with peaks at 1386, 1215, and 1132 cm^−1^ attributed to aromatic C–N and 751, 720, and 692 cm^−1^ aromatic C-S stretchings. In addition, 1189, 1045, 868, and 810 cm^−1^ aromatic S-H stretchings, further support the structure of compound **3** [24,25,26,27].

In the FT-IR spectra of the synthesized compound **4** (Figure S2), the disappearance of the sharp (Ar–C≡N) peak at 2220 cm^−1^ present in compound **3** and the appearance of new bands associated with the phthalocyanine core at 1714 cm^−1^ (Ar–C=N), 1436 cm^−1^ (Ar–C–N), 1377, 1035, and 1001 cm^−1^ (Ar–C=N) provide strong evidence for the formation of the metal-phthalocyanine macrocycle [25,26,27,28].

Only the ^1^H and ^13^C NMR spectra of compound **3** were successfully obtained (Figures S3 and S4), while the phthalocyanine compounds yielded limited information due to their high photocatalytic reactivity, which interferes with clear NMR signal resolution [25,26,27].

**Figure 1:**
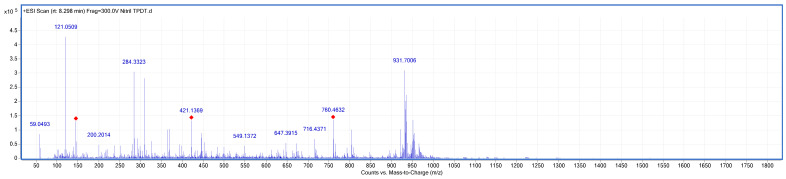
Mass spectrometry (Q-TOF-MS) of compound **3**.

**Figure 2:**
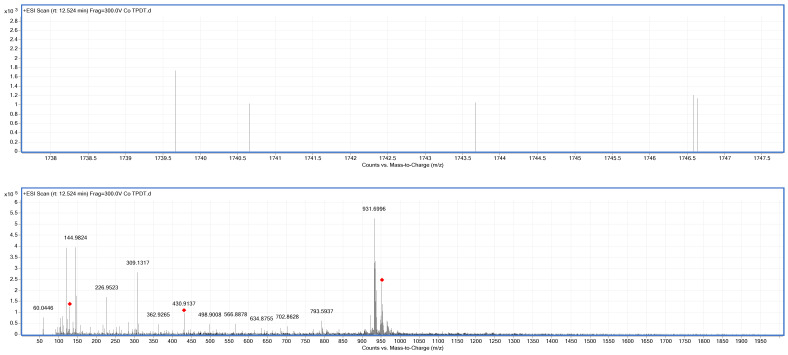
Mass spectrometry (Q-TOF-MS) of compound **4**.

Mass spectrometry (Q-TOF-MS) was therefore employed as a more effective technique for structural elucidation. The spectrum of compound **3** (Figure 1) displayed a protonated molecular ion at *m*/*z* 421.14 ([M+H]^+^), consistent with the calculated value of *m*/*z* 420.08 for C_26_H_16_N_2_S_2_. The Q-TOF-MS spectrum of compound **4** (Figure 2) showed protonated molecular ions at *m*/*z* 1739.20 [M-H]^+^ (calculated *m*/*z* 1741.13 for C_104_H_64_N_8_S_8_Co).

These spectral results confirm the successful synthesis of the target cobalt phthalocyanine derivatives as designed and presented in Scheme 1.

**Figure 3:**
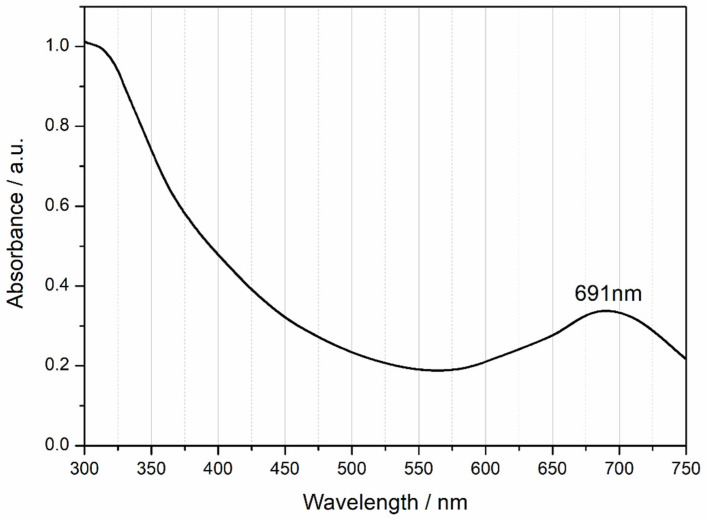
UV–Vis absorption spectra of **4** in DMSO.

The electronic absorption spectra are essential for understanding the optical and electronic properties of phthalocyanines (Pcs). The UV–Vis spectrum of compound **4** in DMSO, as illustrated in Figure 3, exhibits characteristic absorption features. The Q-bands, located in the range of 600–691 nm, are typical for phthalocyanine derivatives and are attributed to π→π* transitions between the highest occupied molecular orbital (HOMO) and the lowest unoccupied molecular orbital (LUMO) of the phthalocyanine macrocycle. Additionally, a B-band absorption appears around 300 nm, which corresponds to transitions from deeper-lying π orbitals to LUMO levels. The observed splitting of the Q-band in both compounds is a well-known hallmark of metal-free or asymmetrically substituted phthalocyanines [3], indicating the electronic asymmetry within the macrocycle.

### 3.2. Computational Results

**Figure 4:**
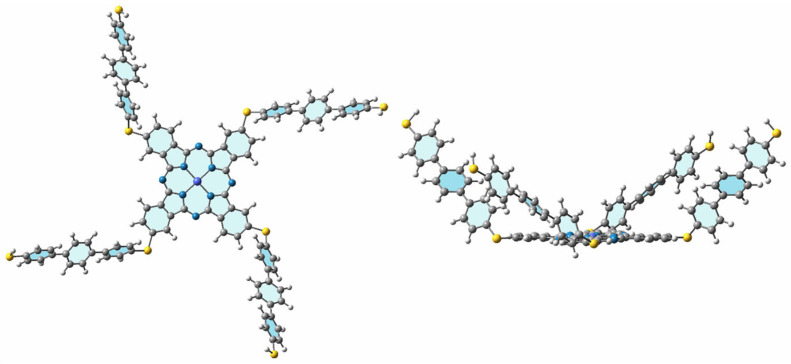
Optimized geometry of compound **4** in DMSO calculated at the B3LYP-D3/6-31G(d) level. (Co: dark blue, N: blue, S: yellow, C: grey; H: white in Gaussview software).

The optimized structures of **4** in ground state are given in Figure 4 in DMSO at the B3LYP-D3/6-31G(d) level. Figure S5 also shows molecular structures with different views of 4 in gas phase. Some important interatomic distances and some selected angles, dipole moments (µ, Debye), total electronic energies including zero-point energy (E_elec_ + ZPE, Hartree), total electronic energies, and thermal free energy corrections (E_elec_ + ΔG, Hartree of **4** calculated with B3LYP-D3 functionals using the 6-31G(d) basis set in gas and solvent DMSO are given in Table S1.

The dipole moment of the complex **4** increases from 4.84 D in the gas phase to 6.77 D in DMSO, suggesting greater charge separation and stabilization in the polar solvent environment. Electronic energy values of the complex is more negative in DMSO compared to the gas phase, indicating that the complex is thermodynamically more stable in the solvent environment.

The metal-nitrogen (Co-N1) and carbon-sulfur (C1-S) bond distances remain unchanged in the gas phase and solvent calculations. The planarity of the phthalocyanine (Pc) core can be assessed using the N-M-N angles. The calculated N-Co-N angle is 179.9°, suggesting that the Pc core remains nearly planar in the complex.

While bond angles remain largely unchanged between the gas-phase and DMSO calculations, notable differences are observed in dihedral angles. The angle between the ligand and the phthalocyanine (Pc) core (C1-S-C2-C3) is 133.66° in the gas phase and decreases to 123.84° in DMSO. This change suggests a solvent-induced structural adjustment, likely influenced by increased dipole moment and enhanced stabilization of a more compact geometry in the polar environment. The phenyl rings in the ligand exhibit a dihedral angle of approximately 36° in the gas phase and 34° in DMSO, indicating only minimal conformational changes upon solvation.

**Figure 5:**
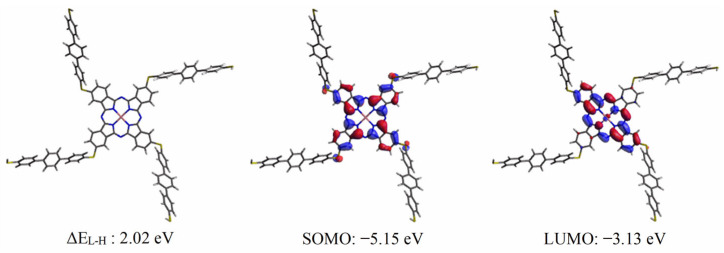
Calculated SOMO and LUMO energies and energy difference of compound **4** in DMSO at the B3LYP-D3/6-31+G(d) level. (Co: pink, N: blue, S: yellow, C: dark gray, H: white in Avogadro software, https://avogadro.cc/).

HOMO and LUMO energies are generally correlated with electron affinity and ionization potential, respectively. Figure 5 displays the energies for SOMO–LUMO and energy gap for **4** in DMSO. Since Co(II) has an unoccupied single electron, the single occupied molecular orbital (SOMO) was shown for **4**. Intramolecular charge transfer occurs much more easily with lower energy gaps. The energy gap between the SOMO and LUMO for compound **4** is 2.02 eV (the small energy gap in compound **4** suggests potential for electronic transitions: a desirable feature in photovoltaic materials). In contrast to our previous study, which examined CoPc with oxygen [28] (where we newly calculated a 2.14 eV value at the same level), this work focuses on CoPc with sulfur (Figure S6). A lower energy gap enhances intramolecular charge transfer, making the process occur much more easily in compound **4**. Additionally, we compared this value with the experimental data and applied the Tauc equation [29]. The optical band gap was determined to be 2.10 eV based on the measured absorption spectrum (Figure S7). This value is in close agreement with the calculated band gap (SOMO-LUMO), which was found to be 2.02 eV.

Weak interactions were analyzed using reduced density gradient (RDG) calculations performed with the Multiwfn program [30,31]. RDG and sign (λ_2_) ρ are a pair of very important functions for revealing weak interaction regions; they are collectively employed in NCI method [32]. The color scale indicates the regions and types of non-covalent interactions. The RDG plot (Figure S8) reveals significant van der Waals interactions and steric effects within the rings, as indicated by the green and red regions, respectively. These interactions may play a role in stabilizing molecular packing and facilitating electronic transitions, which are critical for the design of efficient photovoltaic materials.

Given that compound **4** contains unpaired electrons, spin density mapping was performed to visualize the localization of the radical character and gain insight into its electronic structure. Figure S9 presents the spin density maps of compound **4** in both its neutral and ionic forms. Notably, the ionic form displays no unpaired electron density. Spin density analysis reveals that the unpaired electron in the neutral form is primarily localized on the central cobalt (Co) atom, highlighting the metal-centered radical character of compound **4**. This localization may influence charge transfer processes and electronic transitions, which are important factors for the compound’s photovoltaic performance.

In addition, the Fukui function was employed to identify specific regions or atoms within compound **4** that exhibit electrophilic or nucleophilic behavior [33], providing further insight into its reactive sites relevant to photovoltaic applications. Fukui functions are chemical descriptors used to identify atomic sites susceptible to electrophilic and nucleophilic attacks. The condensed Fukui functions and dual descriptor for the studied compound were calculated using the Multiwfn program based on its N (neutral), N+1 (anion), and N−1 (cation) forms, respectively. Using the finite difference approximation, the Fukui function can be unambiguously calculated for three cases (Figure S10). In the map, green and blue isosurface correspond to positive and negative region of functions, respectively.

Clearly, the most positive regions of the f^−^ (electrophilic) Fukui function are localized on atoms N16 and N26 (surrounding the Co center), as well as on carbon atoms C14 and C128, which are part of the phthalocyanine (Pc) core and phenyl rings (Table S2). These regions are identified as the most susceptible to electrophilic attack due to their pronounced nucleophilic character.

The cobalt (Co) center exhibits a positive f^−^ value, indicating susceptibility to electrophilic attack due to its nucleophilic character. However, the negative f^+^ value suggests that Co is not electrophilic and is unlikely to be targeted by nucleophiles. This combination means that Co can donate electron density (nucleophilic site) but does not readily accept electrons (not electrophilic). Despite the positive NBO charge indicating partial electron deficiency, the Fukui functions reveal that Co acts primarily as a nucleophilic site within the compound. Together with the positive f^−^ and negative f^+^ values, these results suggest that Co primarily acts as a nucleophilic site susceptible to electrophilic attack but is relatively inert toward radical species and nucleophilic attack.

The sulfur atoms in the ligands exhibit mostly positive values for the f^0^ (radical Fukui function) and the dual descriptor, indicating that these sites are more susceptible to radical attacks and show a tendency toward electrophilic behavior. This suggests that sulfur atoms play a significant role in the compound’s overall reactivity, particularly in processes involving radical or electrophilic interactions.

In general, the positive regions (often visualized in green) of an atom’s electron density can be susceptible to electrophilic attack. In the context of quantum chemistry and molecular orbital theory, areas of lower electron density or partial positive charge—such as those near certain atomic centers or within specific molecular orbitals—tend to attract electrophiles. This is particularly important in understanding charge distribution and reactivity patterns in nucleophilic–electrophilic interactions.

**Figure 6:**
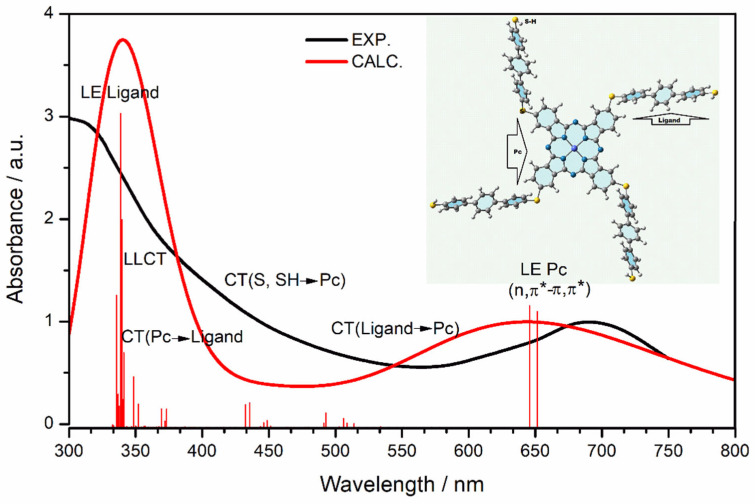
Measured and calculated (B3LYP/6-31+G(d,p)) UV–Vis absorbance spectra of **4** in DMSO.

The absorption wavelengths and excitation energies of all investigated systems from S_0_ to S_120_ states were calculated using TD-DFT at the B3LYP-D3/6-31+G(d,p) level using optimized ground-state geometries. Figure 6 compares the computed and experimentally measured UV–Vis absorption spectra of molecule **4** in DMSO. As seen in the figure, the computational and experimentally measured UV–Vis spectra are in good agreement, demonstrating consistency between the two. Table S3 presents detailed information on the selected electronic transitions, oscillator strengths (f), excitation characteristics, and molecular orbital contributions for molecule **4** in DMSO. The corresponding molecular orbitals associated with these transitions are depicted in Figure S11 in the Supplementary Material.

Q-bands (651.4 nm and 645.7 nm for **4**) were observed with high oscillator strength (LE Pc: locally excited Pc, n-π*, π-π* transition). Molecule **4** exhibits distinct electronic transitions corresponding to charge transfer processes across different wavelength ranges. The charge transfer from the ligand to the Pc occurs between 493 nm and 524 nm, while the transfer from sulfur (S) and thiol (SH) to Pc is observed in the range of 433 nm to 451 nm. Additionally, electronic transitions associated with charge transfer from Pc to the ligand are found between 369 nm and 387 nm. Ligand-to-metal charge transfer (LMCT) and metal-to-ligand charge transfer (MLCT) transitions were observed at 411 nm and 403 nm, respectively, with oscillator strengths close to zero, indicating very weak transitions. At 341 nm, the B band exhibits a locally excited (LE) π,n* and π-π* transition in Pc, characterized by a high oscillator strength. The transitions observed at 340 nm and 339 nm exhibit significantly higher oscillator strengths and involve locally excited (LE) ligand states as well as ligand-to-ligand charge transfer (LLCT).

## 4. Conclusions

The calculated and experimental results for compound **4**, including elemental analysis and FT-IR, ^1^H-^13^C-NMR, and UV–Vis absorption spectra, exhibited strong mutual consistency, confirming the accuracy of both the synthesis and characterization processes. The optical band gap determined from Tauc plot analysis (2.10 eV) closely matched the calculated SOMO–LUMO gap (2.02 eV), confirming the reliability of the computational approach. Reduced density gradient (RDG) analysis revealed significant weak interactions, including van der Waals forces and steric effects, primarily within the phthalocyanine rings, which may influence molecular packing and stability. Spin density mapping demonstrated that the unpaired electron is mainly localized on the cobalt center, indicating a metal-centered radical character crucial for understanding its reactivity. Fukui function calculations further elucidated the reactive sites of compound **4**. The cobalt center shows a nucleophilic character, being susceptible to electrophilic attack, while the sulfur atoms in the ligands exhibited susceptibility to radical and electrophilic interactions. These findings are consistent with the NBO charge analysis, highlighting the complex interplay of charge distribution and reactivity in the molecule.

The electronic transitions observed—encompassing ligand-to-metal, metal-to-ligand, and ligand-to-ligand charge transfer and locally excited states—provide meaningful insight into the electronic structure and photophysical behavior of the molecule. Collectively, these findings enhance our understanding of compound **4**′s structural stability, chemical reactivity, and optoelectronic properties.

In this study, it was observed that phthalocyanine compounds containing sulfur-based ligands exhibit a lower energy gap. Additionally, future work will explore the photoluminescence and solid-state emission properties of these compounds to evaluate their full optoelectronic potential. Compared to those with oxygen-based ligands, leading to more efficient and facile intramolecular charge transfer.

The combined theoretical and experimental insights provide a comprehensive understanding of compound **4**′s electronic and reactive properties, supporting its potential use in photovoltaic devices. Future work could explore the impact of structural modifications on these properties to optimize performance.

## Data Availability

The original contributions presented in this study are included in the article/Supplementary Materials. Further inquiries can be directed to the corresponding author(s).

## Supplementary Materials

The following supporting information can be downloaded at; https://www.mdpi.com/article/10.3390/molecules30132693/s1, Figure S1: FT-IR Spectrum of Compound **3**; Figure S2: FT-IR Spectrum of Compound **4**; Figure S3: 1H-NMR Spectrum of Compound **3**; Figure S4: 13C-NMR Spectrum of Compound **3**; Figure S5: Optimized geometry of **4** in gas phase (atom numbers and symbols were used in Table S1; Co: dark blue, N: blue, S: yellow, C: grey; H: white); Table S1: Some important interatomic distances and some selected angles, dipole moments (µ, Debye), total electronic energies including zero-point energy (E_elec_+ZPE, Hartree), total electronic energies and thermal free energy corrections (E_elec_+ΔG, Hartree) of **4** calculated with B3LYP-D3 functionals using the 6-31G(d) basis set in gas and solvent DMSO; Figure S6: Calculated SOMO, LUMO energies and energy differences of compound **4** (A) and Cobalt Phthalocyanine (Co-Pc) **5** (B) in DMSO at B3LYP-D3/6-31+G(d) level; Figure S7: Tauc plot of **4** in DMSO (Eg = 2.10 eV); Figure S8: Plots of the product of the electron density Hessian matrix (sign of λ_2_) and the electron density (ρ) with the reduced density gradient (RDG) for investigated systems. The default RDG isosurface is 0.5 and the color range is −0.035 to 0.02. (Calculated at B3LYP-D3/6-31G(d), NBO); Figure S9: Spin density maps of compound **4** in its neutral and ionic forms; Figure S10: Electron density maps from f^−^, f^+^, f^0^ and DD calculations (isovalue: 0.007 a.u.); Table S2: NBO charges of N (neutral), N+1 (anionic), N-1 (cationic) states, fukui functions and dual descriptors of selected atoms in compound **4** in DMSO; Table S3: Selected parameters for the vertical excitation of **4** (CoPc) predicted by the TD-DFT at B3LYP-D3/6-31+G(d) level in DMSO (Electronic transitions (λ_ex_) corresponding to vertical excitation energies (ΔE), oscillator strengths (f), excitation character, molecular orbitals and their % contributions, S: SOMO, L: LUMO); Figure S11: Selected molecular orbitals of 4 in DMSO at B3LYP-D3/6-31+G(d) levels (isovalue is 0.02 a.u.).
