# Supplementary material for: Structure–Function Relationship of Novel Tetrakis (Mercapto-Terphenyl)Benzene Cobalt (II) Phthalocyanines: Synthesis and Computational Evaluation
Source: Molecules. 2025 Jun 22;30(13):2693. doi: 10.3390/molecules30132693 (PMC12251080; doi:10.3390/molecules30132693)
Supplement: Supplementary file 1 [file molecules-30-02693-s001.zip › molecules-3701075-supplementary.pdf]

Electronic Supplementary Material

Structure–Function Relationship of Novel Tetrakis (Mercapto-Terphenyl)Benzene Cobalt (II) Phthalocyanines: Synthesis and Computational Evaluation

Sevil Sener [1,*] and Nursel Acar-Selcuki [2]

[1] Department of Chemical Technology Program, Aliaga Vocational School, Ege University, Izmir 35040, Türkiye
[2] Department of Chemistry, Faculty of Science, Ege University, Izmir 35100, Türkiye; nursel.acar@ege.edu.tr
* Correspondence: sevil.sener@ege.edu.tr

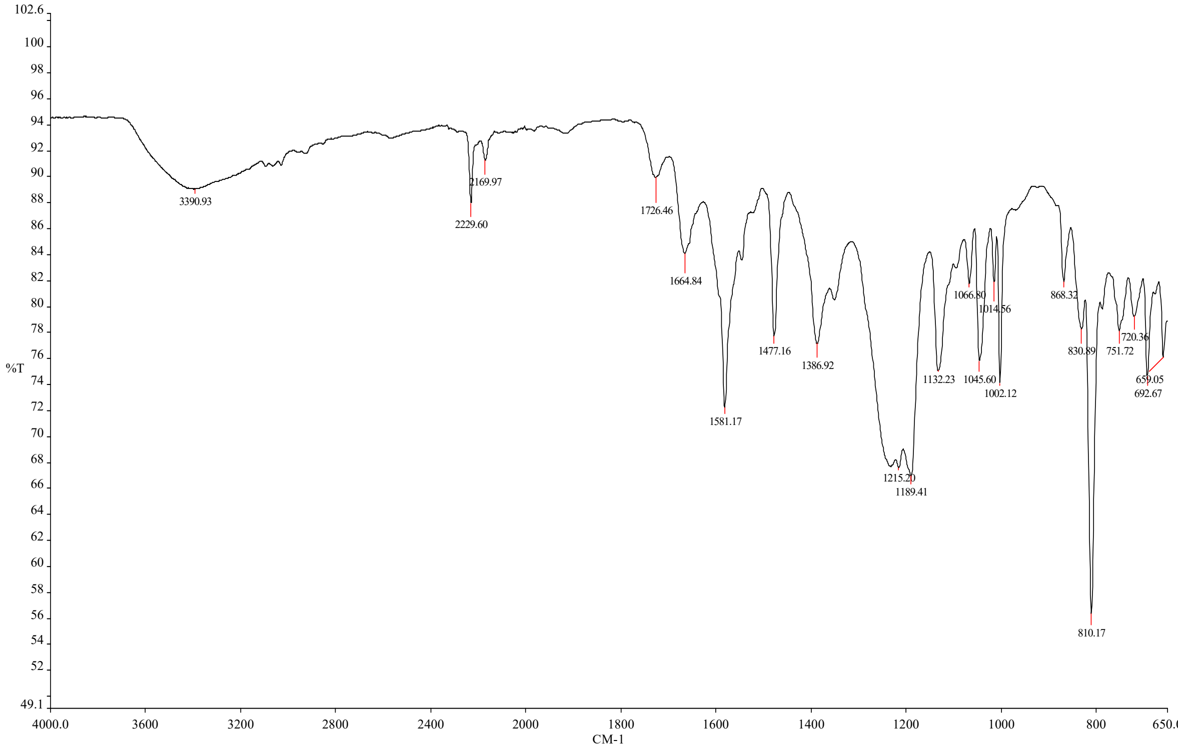

Figure S1. FT-IR Spectrum of Compound **3**

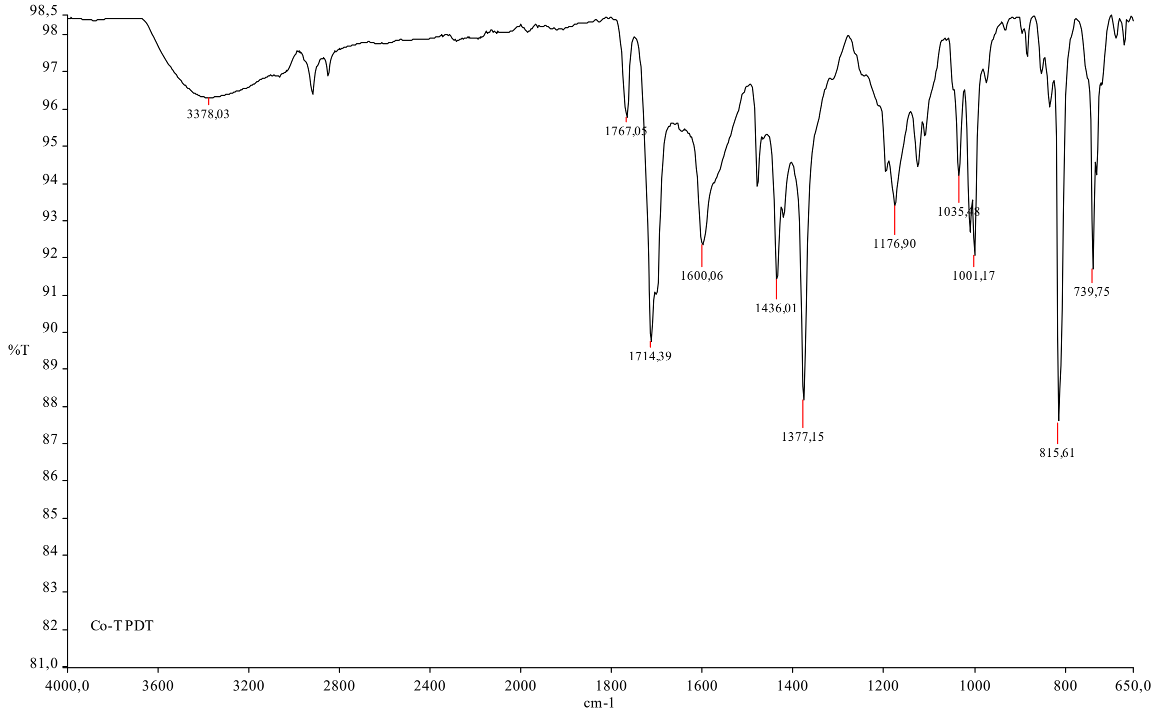

Figure S2. FT-IR Spectrum of Compound **4**

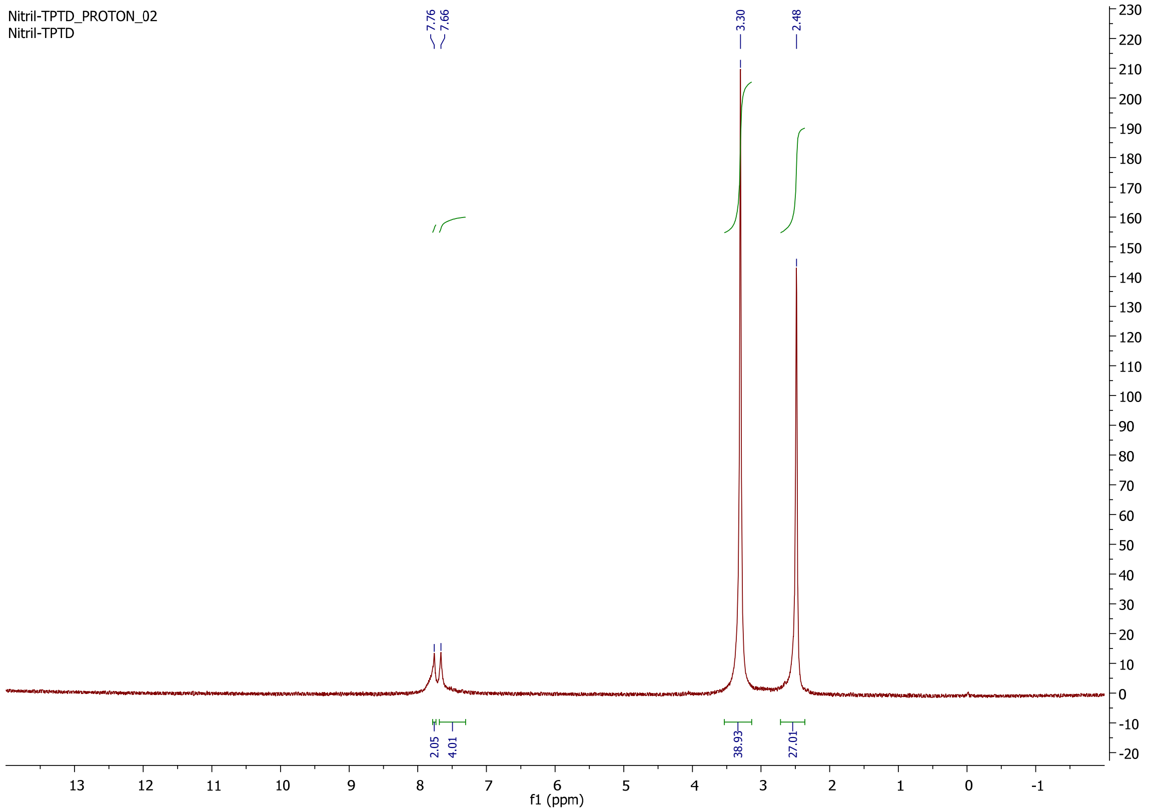

Figure S3. ^{1}H-NMR Spectrum of Compound **3**

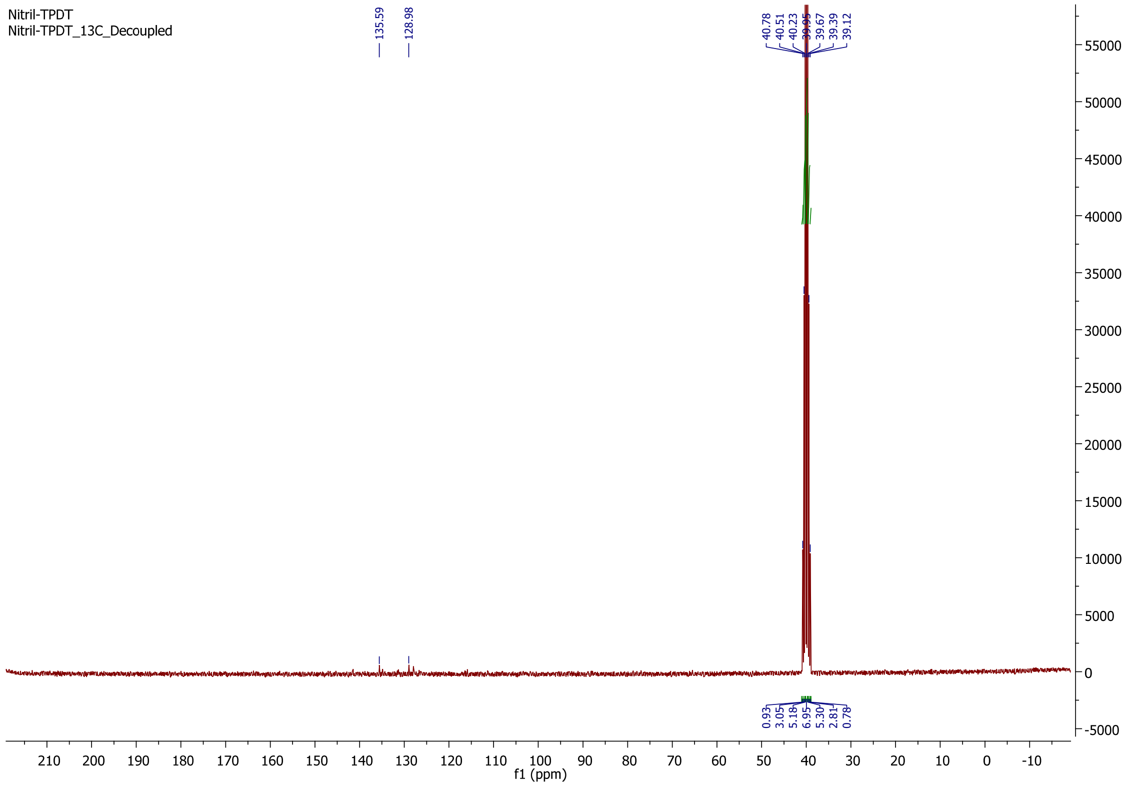

Figure S4. ^{13}C-NMR Spectrum of Compound **3**

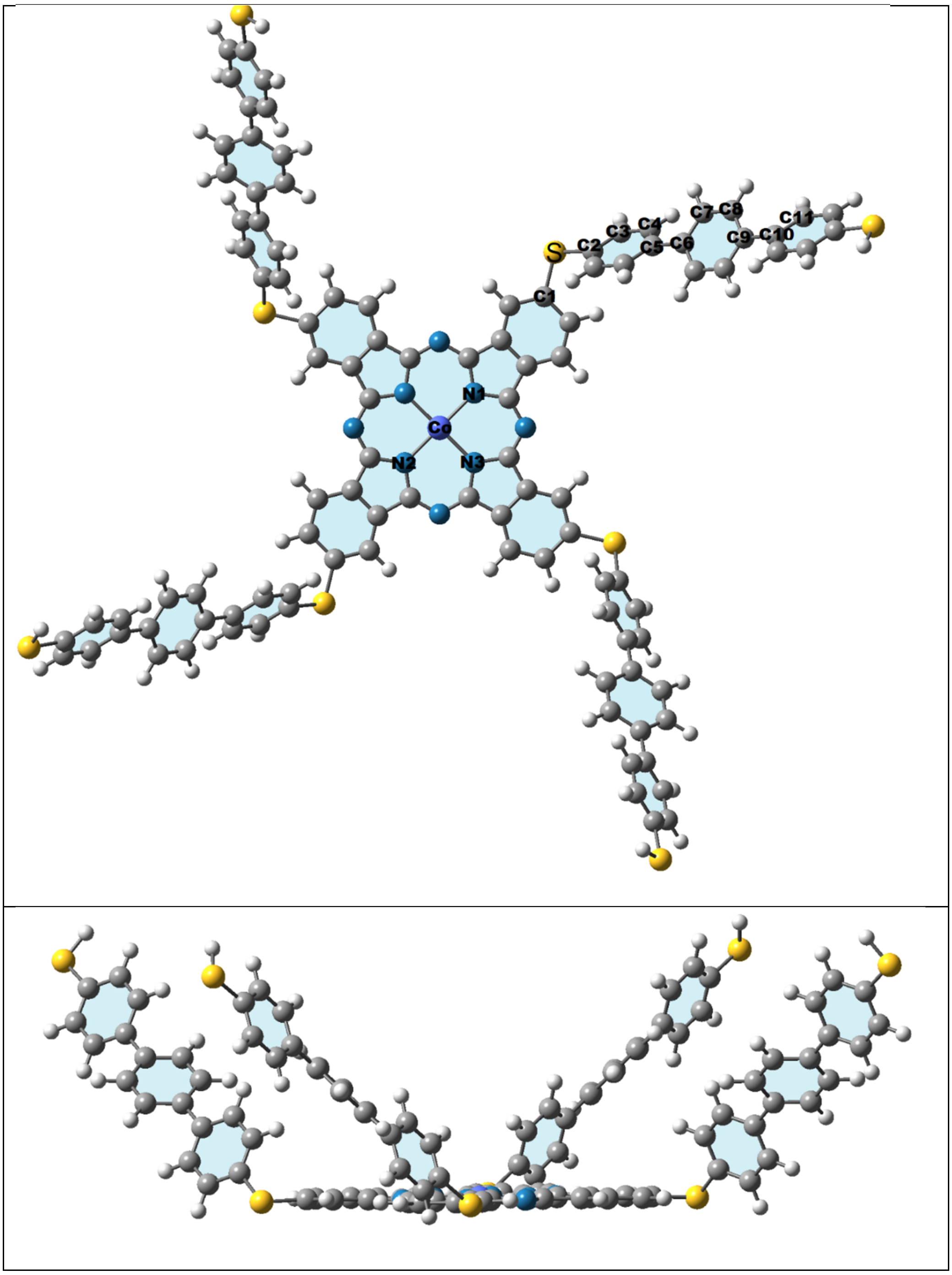

Figure S5. Optimized geometry of **4** in gas phase (atom numbers and symbols were used in Table S1; Co: dark blue, N: blue, S: yellow, C: grey; H: white)

Table S1. Some important interatomic distances and some selected angles, dipole moments (μ, Debye), total electronic energies including zero-point energy (E_{elec}+ZPE, Hartree), total electronic energies and thermal free energy corrections ($E_{elec}+\Delta G$, Hartree) of **4** calculated with B3LYP-D3 functionals using the 6-31G(d) basis set in gas and solvent DMSO

	**Gas**	**DMSO**
μ (D)	4.84	6.77
E_{elec}+ZPE (Hartree)	−9007.1971	−9007.2322
$E_{elec}+\Delta G$ (Hartree)	−9007.3667	−9007.3970
Freq (cm^{-1})	0.97	1.95
**distances (Å)**	**Gas**	**DMSO**
Co-N1	1.92	1.92
C1-S	1.79	1.79
**bond angles [º]**	**Gas**	**DMSO**
N1-Co-N2	179.91	179.96
N3-Co-N1	90.00	89.95
C1-S-C2	102.30	102.79
S-C2-C3	118.91	119.30
C4-C5-C6	121.09	121.00
C8-C9-C10	121.15	121.13
**dihedral angles [º]**	**Gas**	**DMSO**
C1-S-C2-C3	133.66	123.84
C4-C5-C6-C7	35.79	34.19
C8-C9-C10-C11	−35.60	−34.05

	SOMO (eV)	LUMO (eV)	ΔE_{L-H}
A	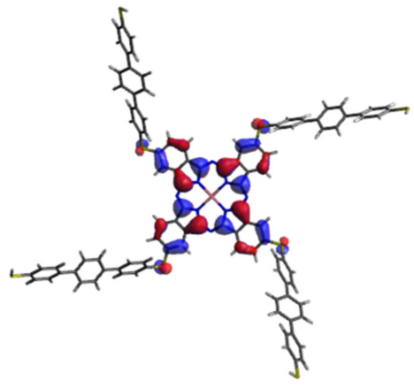 −5.15	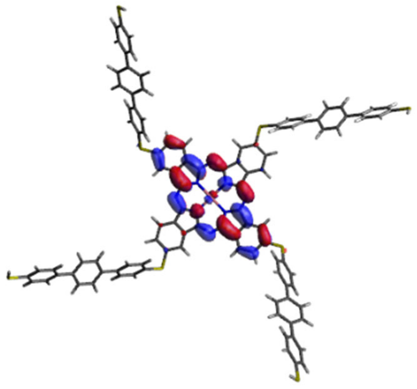 −3.13	**2.02**
B	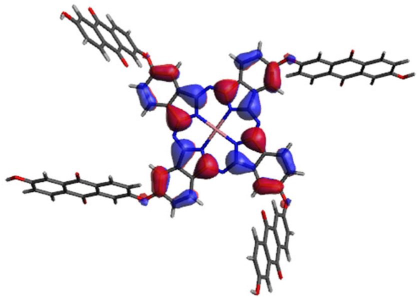 −5.37	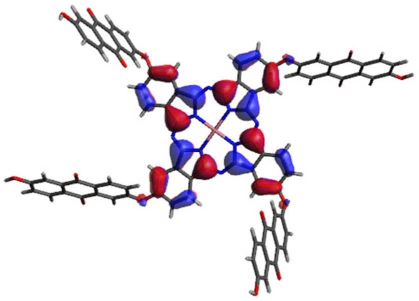 −3.23	2.14

Figure S6. Calculated SOMO, LUMO energies and energy differences of compound **4** (A) and Cobalt Phthalocyanine (Co-Pc) **5** (B) in DMSO at B3LYP-D3/6-31+G(d) level.

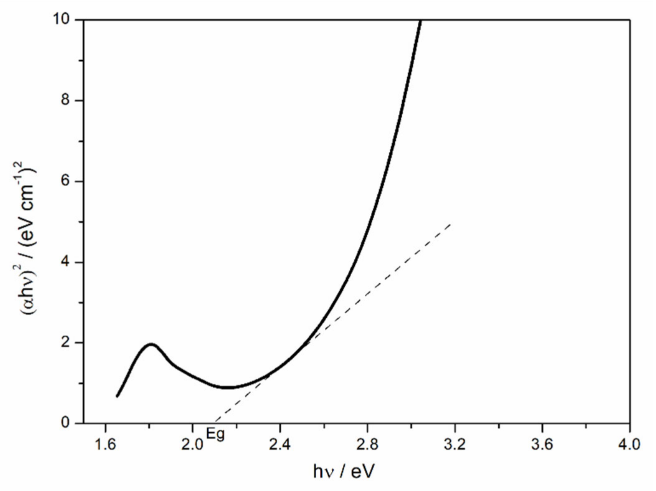

Figure S7. Tauc plot of **4** in DMSO (Eg = 2.10 eV)

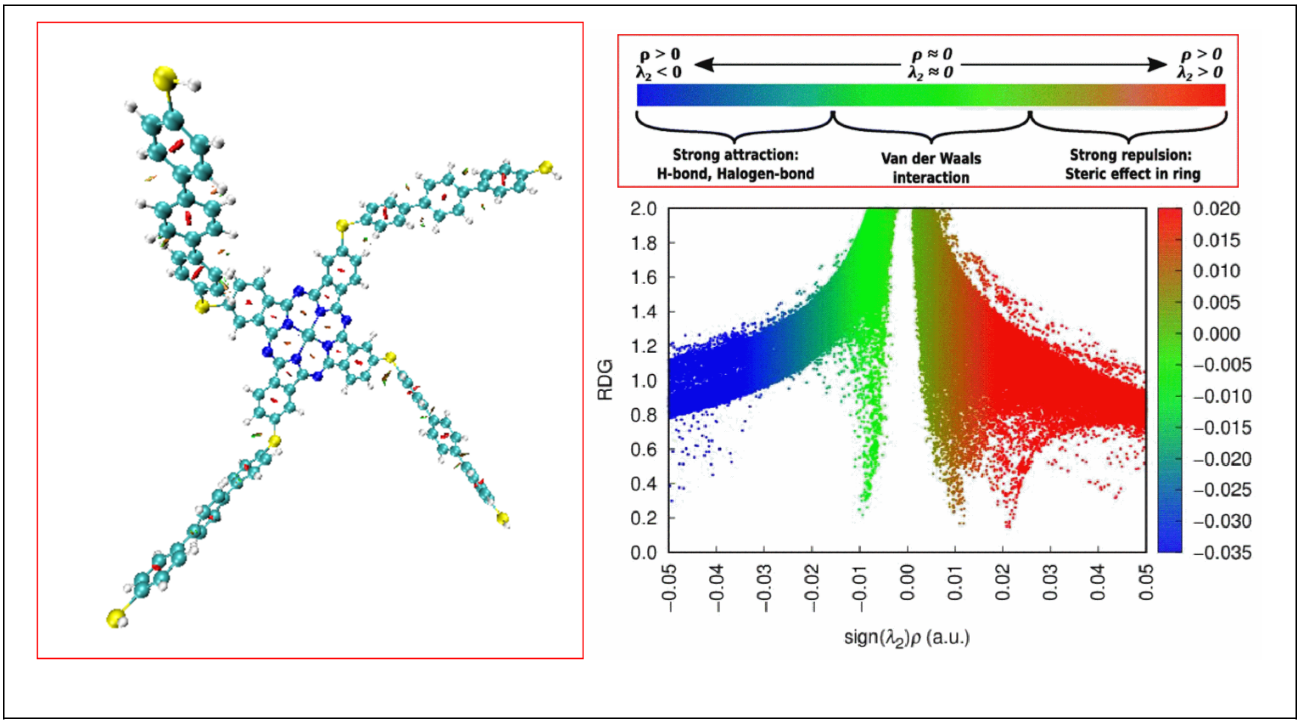

Figure S8. Plots of the product of the electron density Hessian matrix (sign of λ_2) and the electron density (ρ) with the reduced density gradient (RDG) for investigated systems. The default RDG isosurface is 0.5 a.u. and the color range is -0.035 to 0.02. (Calculated at B3LYP-D3/6-31G(d), NBO)

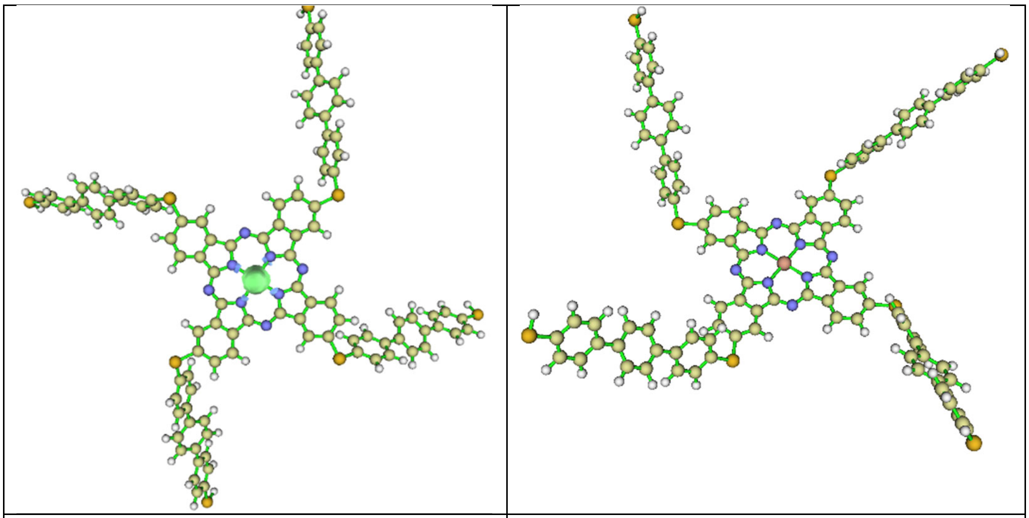

Neutral form	Ionic form

Figure S9. Spin density maps of compound **4** in its neutral and ionic forms.

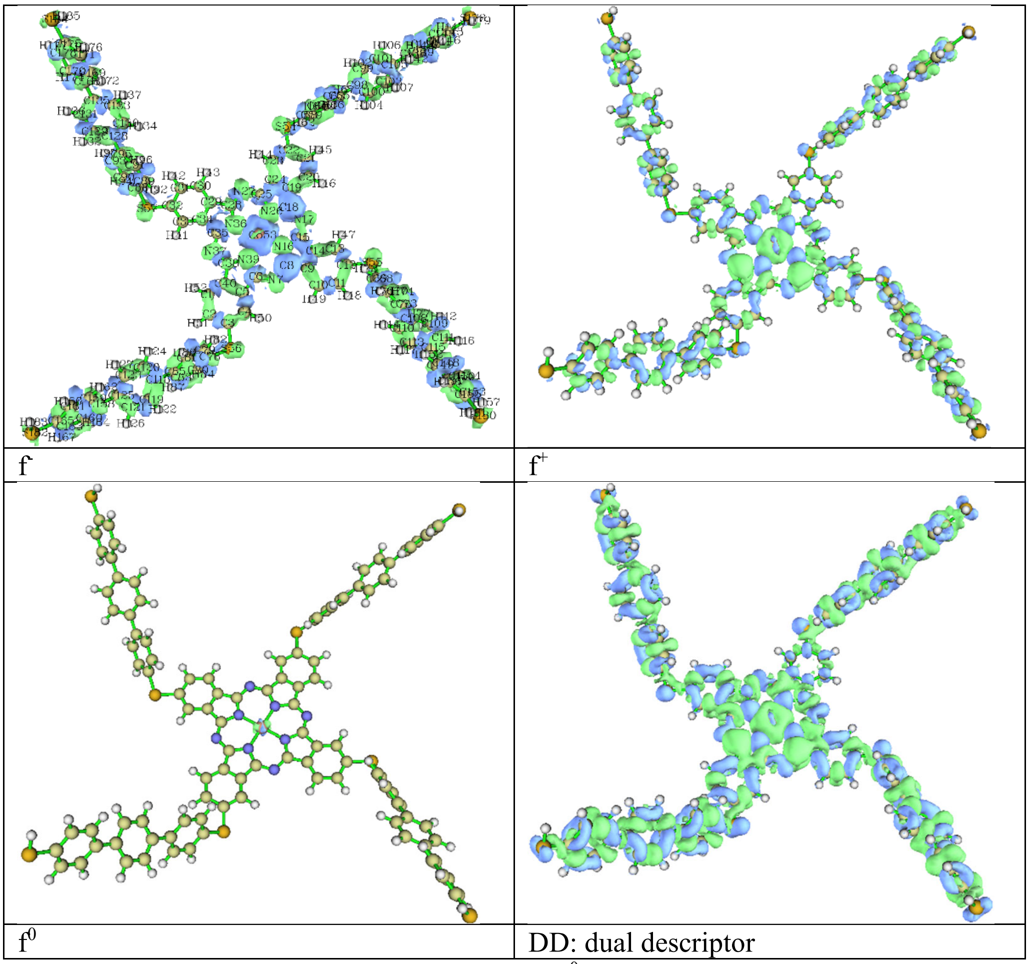

Figure S10. Electron density maps from f- , f+, f^0 and DD calculations (isovalue: 0.007 a.u.)
Nucleophilic attack (blue): $f^+(r) = \rho_{N+1}(r) - \rho_N(r) \approx \rho^{LUMO}(r)$;
Electrophilic attack (green) : $f^-(r) = \rho_N(r) - \rho_{N-1}(r) \approx \rho^{HOMO}(r)$;
Radical attack: : $f^0(r) = 1/2\,[f^+(r) + f^-(r)] = 1/2\,[\rho_{N+1}(r) - \rho_{N-1}(r)] \approx \frac{1}{2}\,[\rho^{HOMO}(r) + \rho^{LUMO}(r)]$
$+ \rho_{N+1}(r) - \rho_N(r) \approx \rho^{LUMO}(r)$, DD = $f^+(r) - f^-(r)$, where ρ stands for the population of an atom in the molecule.

Table S2. NBO charges of N (neutral), N+1 (anionic), N-1 (cationic) states, fukui functions and dual descriptors (DD) of selected atoms in compound **4** in DMSO

Atom	N	N+1	N-1	f^+	f^-	f^0	DD
Co53	0.97348	0.71584	0.95573	-0.25764	0.01775	-0.1377	-0.27539
N16	-0.60054	-0.58725	-0.60384	0.01329	0.0033	0.00499	0.00999
N26	-0.60063	-0.58776	-0.60081	0.01287	1.8E-4	0.00635	0.01269
N36	-0.60054	-0.58725	-0.59161	0.01329	-0.00893	0.01111	0.02222
N39	-0.60063	-0.58776	-0.59419	0.01287	-0.00644	0.00966	0.01931
C8	0.42238	0.38986	0.47209	-0.03252	-0.04971	0.0086	0.01719
C18	0.44135	0.39106	0.53201	-0.05029	-0.09066	0.02018	0.04037
S54	0.31911	0.30116	0.39651	-0.01795	-0.0774	0.02972	0.05945
S55	0.31309	0.29996	0.33428	-0.01313	-0.02119	0.00403	0.00806
S56	0.31912	0.30117	0.32393	-0.01795	-0.00481	-0.00657	-0.01314
S57	0.3131	0.29997	0.35294	-0.01313	-0.03984	0.01336	0.02671
C12	-0.20208	-0.21119	-0.19795	-0.00911	-0.00413	-0.00249	-0.00498
C13	-0.20169	-0.21638	-0.16092	-0.01469	-0.04077	0.01304	0.02608
C14	-0.07707	-0.07708	-0.08864	-1E-5	0.01157	-0.00579	-0.01158
C128	-0.05981	-0.0589	-0.06106	9.1E-4	0.00125	-1.7E-4	-3.4E-4
C130	-0.21573	-0.21622	-0.21425	-4.9E-4	-0.00148	4.95E-4	9.9E-4
S178	0.01144	0.01108	0.01299	-3.6E-4	-0.00155	5.95E-4	0.00119
S180	0.01136	0.011	0.01184	-3.6E-4	-4.8E-4	6E-5	1.2E-4
S182	0.01144	0.01108	0.01168	-3.6E-4	-2.4E-4	-6E-5	-1.2E-4
S184	0.01136	0.011	0.01253	-3.6E-4	-0.00117	4.05E-4	8.1E-4
H185	0.16125	0.16122	0.16137	-3E-5	-1.2E-4	4.5E-5	9E-5

Table S3. Selected parameters for the vertical excitation of **4 (CoPc)** predicted by the TD-DFT at B3LYP-D3/6-31+G(d) level in DMSO (Electronic transitions (λ_{ex}) corresponding to vertical excitation energies (ΔE), oscillator strengths (f), excitation character, molecular orbitals and their % contributions, S: SOMO, L: LUMO).

state	ΔE (eV)	λ_{ex} (nm)	f	Predominant Transitions (%)	Character[a]
S8	1.90	651.4	0.85	S → L (68)	LE Pc (n,π*; π,π*)
S9	1.92	645.7	0.88	S → L+1 (68)	LE Pc (n,π*; π,π*)
S18	2.37	523.6	0.0003	S-3 → L+1 (68)	CT1 (ligand→Pc)
S21	2.41	514.1	0.027	S-2 → L (64)	CT1 (ligand→Pc)
S22	2.44	508.8	0.030	S-2 → L+1 (81)	CT1 (ligand→Pc)
S26	2.52	492.9	0.11	S-4 → L (57)	CT1 (ligand→Pc)
S37	2.75	451.4	0.008	S-8 → L (60)	CT (S, SH→Pc)
S42	2.78	446.3	0.032	S-7 → L+1 (68)	CT (S, SH→Pc)
S46	2.84	434.8	0.18	S-8 → L (63)	CT (S, SH→Pc)
S48	2.87	432.6	0.17	S-8 → L+1 (59)	CT (S, SH→Pc)
S56	3.02	410.9	0.0001	S → L+7 (26)	LMCT, LE Pc
S58	3.07	403.4	0.0007	S-10 → L (63)	MLCT, CT1
S66	3.20	387.0	0.002	S → L+2 (63)	CT2 (Pc→ligand)
S74	3.32	373.1	0.13	S → L+3 (73)	CT2 (Pc→ligand)
S75	3.33	371.9	0.044	S → L+4 (70)	CT2 (Pc→ligand)
S77	3.36	369.3	0.13	S → L+5 (77)	CT2 (Pc→ligand)
S110	3.63	341.1	0.54	S→ L+10 (53)	LE Pc (n,π*; π,π*)
S112	3.65	339.7	1.52	S-2 → L+5(32)<br>S-1 → L+4 (31)<br>S → L+11(27)	LE Ligand<br>LE Ligand, LLCT<br>LE Pc (n,π*; π,π*)
S113	3.66	338.6	2.29	S-3 → L+2 (38)<br>S-2 → L+3 (14)	LE Ligand<br>LLCT Ligand

[a]LE Pc: locally excited Pc; LE Ligand: locally excited ligand; CT1: intramolecular charge transfer from ligand to Pc; CT2: intramolecular charge transfer from Pc to ligands; CT: intramolecular charge transfer from Pc to S, SH; LMCT: charge transfer from metal to ligands; MLCT: charge transfer from ligand to metal; LLCT: charge transfer between ligands

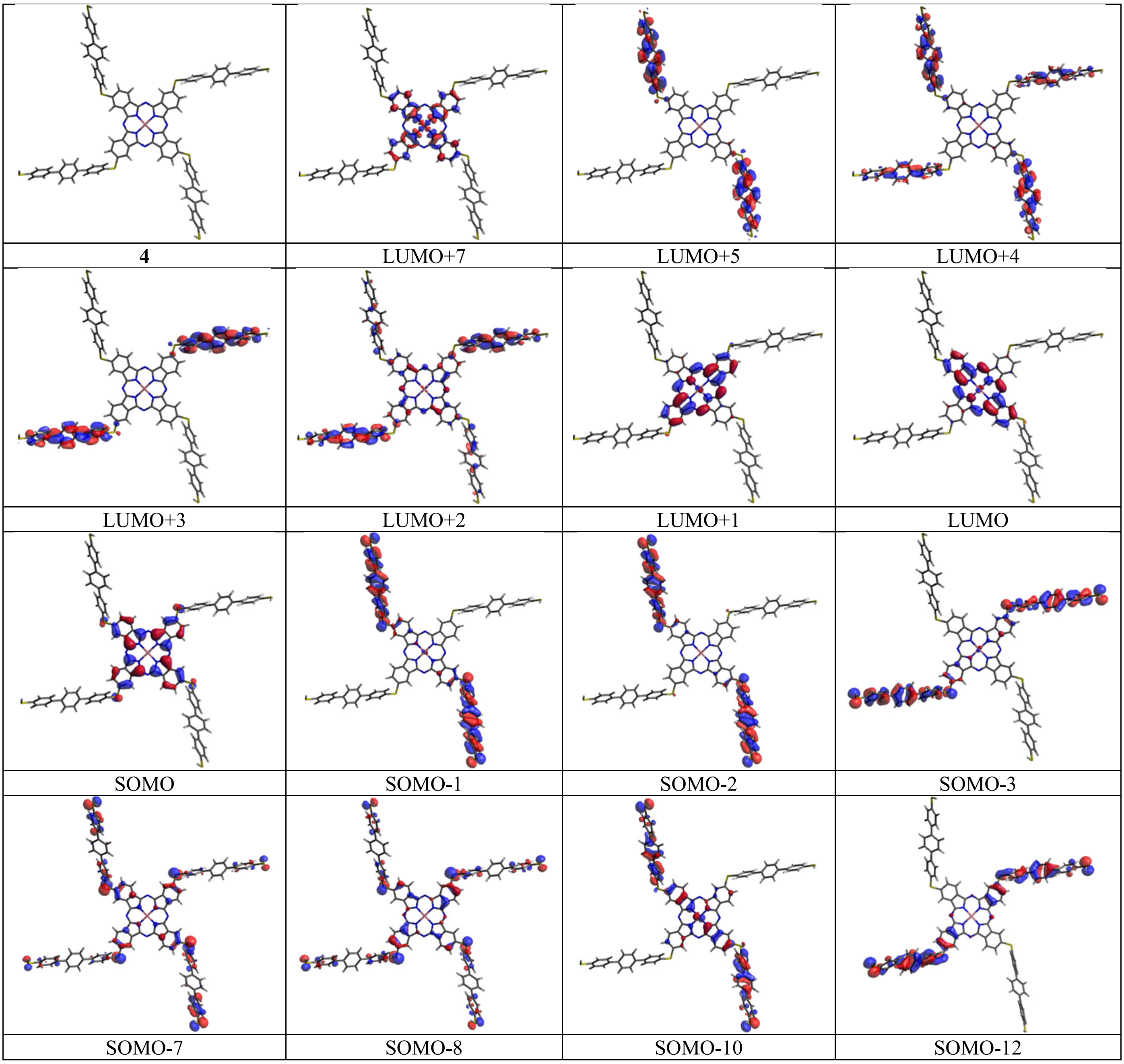

Figure S11. Selected molecular orbitals of 4 in DMSO at B3LYP-D3/6-31+G(d) levels (isovalue is 0.02 a.u.).
